# Structural Characterization of the Interaction of Hypoxia Inducible Factor-1 with Its Hypoxia Responsive Element at the −964G > A Variation Site of the *HLA-G* Promoter Region

**DOI:** 10.3390/ijms222313046

**Published:** 2021-12-02

**Authors:** Cinthia C. Alves, Eduardo A. Donadi, Silvana Giuliatti

**Affiliations:** 1 Department of Genetic, Ribeirão Preto Medical School, University of São Paulo, Ribeirão Preto 14049-900, Brazil; cinthiacalves@alumni.usp.br; 2 Department of Medicine, Ribeirão Preto Medical School, University of São Paulo, Ribeirão Preto 14049-900, Brazil; eadonadi@fmrp.usp.br

**Keywords:** hypoxia induced factor-1, hypoxia regulatory element, HLA-G, protein-DNA interaction, molecular docking

## Abstract

Human Antigen Leukocyte-G (*HLA-G*) gene encodes an immune checkpoint molecule that has restricted tissue expression in physiological conditions; however, the gene may be induced in hypoxic conditions by the interaction with the hypoxia inducible factor-1 (HIF1). Hypoxia regulatory elements (HRE) located at the *HLA-G* promoter region and at exon 2 are the major HIF1 target sites. Since the G allele of the −964G > A transversion induces higher HLA-G expression when compared to the A allele in hypoxic conditions, here we analyzed HIF1-HRE complex interaction at the pair-atom level considering both −964G > A polymorphism alleles. Mouse HIF2 dimer crystal (Protein Data Bank ID: 4ZPK) was used as template to perform homology modelling of human HIF1 quaternary structure using MODELLER v9.14. Two 3D DNA structures were built from 5′GCRTG’3 HRE sequence containing the −964G/A alleles using x3DNA. Protein-DNA docking was performed using the HADDOCK v2.4 server, and non-covalent bonds were computed by DNAproDB server. Molecular dynamic simulation was carried out per 200 ns, using Gromacs v.2019. HIF1 binding in the HRE containing −964G allele results in more hydrogen bonds and van der Waals contact formation than HRE with −964A allele. Protein-DNA complex trajectory analysis revealed that HIF1-HRE-964G complex is more stable. In conclusion, HIF1 binds in a more stable and specific manner at the HRE with G allele.

## 1. Introduction

Hypoxia plays an important role in physiological processes, such as embryogenesis and placental development [1,2], but also in pathological conditions, mainly in solid malignant tumors [3,4,5,6]. Several studies have shown that hypoxia is strongly associated with lower overall survival and disease-free survival of several tumor types [3,4,6]. A decreased oxygen concentration in the tumor micro-environment leads to an adaptive response with modulation of gene expression involved in glycolysis, erythropoiesis, angiogenesis, and in many immune system regulators, facilitating cell survival and immune system evasion [6,7,8,9].

The master regulator of gene expression control in hypoxia is the hypoxia inducible factor 1 (HIF1), a heterodimeric protein that belongs to the basic helix-loop-helix PER-ARTN-SIM (bHLH-PAS) family [10]. As a major regulator of oxygen homeostasis, HIF1 also contributes to the growth of tumors and its increased expression has been associated with poor prognosis. Therefore, the understanding of gene regulation by HF1 has been considered as a potential therapeutic target [6]. The HIF1 heterodimer is formed by an α subunit (HIF1a), whose stability is oxygen-regulated, and a stable β subunit (ARNT). In both subunits, the N-terminal domain (bHLH-PAS-A/B-PAC) is responsible for dimerization and DNA binding, whereas the C-terminal domains are required for protein stabilization and transactivation [11,12,13,14]. In the presence of cellular oxygen, HIF1a is hydroxylated by prolyl-hydroxylases (PHD) and by a factor Inhibitor of HIF1 (FIH), resulting in proteasomal degradation [14,15,16,17,18,19] and coactivator inhibition, such as CBP/p300, which is necessary to target gene transcription activation [19,20]. In contrast, the hydroxylases (PHD and FIH) become nonfunctional and the stabilized HIF1a can translocate into the nucleus to bind ARNT under hypoxia conditions [12]. The formed heterodimeric HIF1 transcription factor recognizes and binds to the hypoxia responsive elements (HREs) at the 5′RCGTG’3 sequence into hypoxia responsive genes to activate transcription process in association with CBP/p300 [21].

During hypoxia, HIF1 induces transcription of several genes that encode proteins involved in the regulation of the immune system [6]. The immune checkpoint human leukocyte antigen-G (*HLA-G*) gene is modulated in hypoxic conditions through HIF1 [22,23]. The membrane-bound HLA-G and the soluble isoforms inhibit the function of several leukocytes, including antigen-presenting (macrophages and dendritic cells), T (CD4 and CD8), B, and Natural Killer (NK) cells, among others, by the interaction with the specific inhibitory receptors present on the leukocyte surface [24,25,26,27]. Physiologically, HLA-G plays a key rule at the fetal-maternal interface keeping immune tolerance due the inhibition of the cytotoxic activity of the maternal TCD8+ and NK cells [28]. However, *HLA-G* may be induced in numerous malignant tumors, such as glioblastoma and melanoma tumors, contributing to tumor immune escape [29].

Since microenvironment hypoxia is an important physiological mechanism for placentation and maternal fetal interface formation, hypoxia has also been associated with increased *HLA-G* expression [30]. Additionally, hypoxia induces *HLA-G* transcription and protein production in a series of HLA-G-negative tumor lineages [22,31,32]. In silico analysis of the 1.4 kb region upstream the ATG translation initiation site revealed the presence of two HREs at positions −242 to −238 bp (nonfunctional) and at positions −966 to −962 positions [23,31,33], which contains a G > A polymorphism at position −964 (rs1632947) [34]. Yaghi et al. described for the first time the impact of the *HLA-G* gene variability at the HIF1-HRE interaction sites in association with the *HLA-G* expression under hypoxic conditions. The authors observed that a glioma tumor cell line that does not express *HLA-G*, when treated with the hypoxia-mimicking agent desferrioxamine (DFX), expressed high amounts of *HLA-G* mRNA and protein. The interaction of HIF1 with HRE at exon 2 presented synergy with the HRE at the promoter (−966 to −962) region, and the *HLA-G* expression was increased in presence of the −964G allele when compared to the −964A allele [23].

Considering that (i) the interaction of proteins with DNA is a crucial event for many cellular processes such as transcription and (ii) the promoter region −964G > A polymorphism affects the transcription of the *HLA-G* gene in hypoxic conditions [23], we evaluated the influence of the HIF1 binding at the *HLA-G* −964G > A polymorphic site at an atom-pair interaction level [35]. This strategy improved the understanding of the differential *HLA-G* regulation by HF1 by observing the physical interaction between the transcription factor and the polymorphism present at the *HLA-G* promoter region regulatory element. 

## 2. Results and Discussion

### 2.1. HIF1 Heterodimer 3D Structure Modeling

We investigated the differential DNA-binding mechanism of HIF-1 into HRE at the *HLA-G* promoter region, considering the −964G > A variation site. Since proteins and DNA molecules differ from their geometry in unbound and bond forms [36], the template chosen for modeling must be a protein-DNA complex, in which the protein has at least 30% of identity to HIF1. Thus, the template chosen for molecular modeling was the crystal of the mouse HIF2 (HIF2a-ARNT-HRE complex) transcription factor, which is a protein belonging to the same protein family as HIF1, which also interacts with HRE [10]. In this crystal, chain A refers to the β subunit (ARNT), while chain B refers to the α subunit (HIF2a). In addition, HIF1 and HIF2 heterodimers have closely related architectures, as point mutations located at the binding sites that destabilize the HIF2 complex also destabilized HIF1 [37]. Furthermore, the global alignment between the α and β subunits of HIF1 and HIF2 revealed that the β subunits share 97.1% of identity and 99% of similarity and the α subunits share 64.8% of identity and 81.4% of similarity between these transcription factors (Supplementary material, Figure S1A,B, respectively), which makes HIF2 a good choice as a template for homology molecular modeling. This crystal has long sequences of missing amino acids (13 to 31 residues) in the tertiary structure of the two subunits, ARNT and HIF2a, which were filled before the HIF1 modeling procedure.

Once the complete tertiary structure of the mouse HIF2 was obtained, it was possible to proceed with the molecular modeling of the human HIF1 transcription factor. For structural modeling, the primary structures of the two HIF1 protein subunits, human HIF1a and ARNT, were recovered from the UniProt database (http://uniprot.org, accessed on 20 January 2020) along with the residues corresponding to the bHLH domains, PAS-A/B and PAC (Residues 15–349 for HIF1a and residues 87–470 for ARNT), which were subsequently aligned with the primary sequence of the 4ZPK crystal chains for modeling, using MODELLER v.9.24 [38,39]. Ten different models were generated and analyzed for their structural similarity with crystal template by calculating the RMSD of protein Cα atom superposition in the PyMol v.2.24 software using align command with five cycles of outlier rejection. The model 8 presented the most similar fold to the 4ZPK crystal with the lowest RMSD value of 0.39 Å (Supplementary material, Figure S2) as compared to the other models. The align command was chosen to perform this analysis because it usually works well for homologous structures with more than 30% of identity, once our structures present more than 60% of identity (Supplementary material, Figure S1). However, when we align both 3D structures without outlier rejection (cycles = 0), the all-atom RMSD value is 6.29 Å, which is a higher value to structural similarity evaluation. However, when we align protein motifs against each other in the model 8 and the 4ZPK crystal using PyMol align command without outlier rejection, RMSD values decrease considerably: bHLH ARNT domains alignment presented a RMSD of 0.64 Å; PAS-A ARNT domains alignment presented a RMSD of 0.75 Å; PAS-B ARNT domains alignment presents a RMSD of 0.82 Å; PAC ARNT domains presented a RMSD of 0.69 Å; bHLH HIF1/2a domains alignment presented a RMSD of 0.71 Å; PAS-A HIF1/2a domains alignment presented a RMSD of 0.96 Å; PAS-B HIF1/2a domains alignment presented a RMSD of 0.69 Å; and PAC HIF1/2a domains alignment presented a RMSD of 0.65 Å (Supplementary material, Figure S3). According to these RMSD values resulting from each domain alignment, we consider that model 8 has a good structural similarity with the template. The higher RMSD value observed before to the all-atom alignment (RMSD: 6.29 Å) could be due loops regions which were allowed to move during the global refinement stage of the MODELLER by a short molecular dynamic simulation, resulting in different model loops conformations in relation to the crystal. Then, this model was refined for loops with MODELLER v.9.24 [40] and evaluated regarding chemical, physical, and stereochemical quality by MQAPs. Quality assessment results obtained to the transcription factor HIF1 (model 8) in comparison with its template are shown in Table 1.

**Figure 1:**
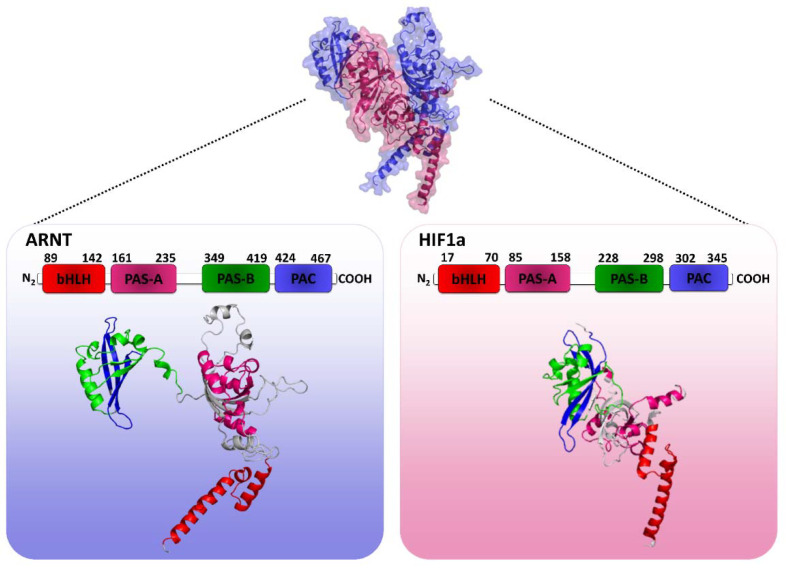
3D representation of the human HIF1 quaternary structure modeling (HIF1a-ARNT complex). Chain A is highlighted in reference to β subunit (ARNT) in blue and chain B is highlighted in reference to α subunit (HIF1a) in pink. Boxes are the structures of each separate chain with illustrated protein domains. Note: Author’s own image.

In general, the quality assessment shows that the model generated by homology for HIF1 showed good physical and chemical quality for most MQAPs in relation to the crystal. In particular, the Ramachandran plot indicates the most favorable, additionally allowed, generously allowed, and disallowed regions for the phi and psi torsion angles. Such analysis is performed for all amino acids, except for glycine and proline because they do not present these angles due to their side chain composition. HIF1 model presented 87.20% of its amino acids in most favorable regions, and 0.80% of amino acids in disallowed regions. Although this model has not an ideal number of amino acids in most favorable and unfavorable regions (above 90% and 0%, respectively), it showed greater stereochemical quality compared to the crystal. However, none of the residues involved in intermolecular interaction with HRE showed torsion angles distribution in generously allowed, and unfavorable regions. Since (i) the present work aims to analyze the structural interaction of DNA with the HIF1 dimer and (ii) residues that bind to the HRE (DNA) have not presented poor quality regarding phi-psi torsion angles arrangement that may affect the binding of the protein complex with DNA, the model generated by homology modeling represented the HIF1 3D structure for carrying out the docking, as presented in Figure 1.

### 2.2. DNA Modeling

The HRE consensus sequence (5′GCGTG’3) at the *HLA-G* promoter region along with fourteen 5′ and 3′ extremity flanking bases was selected to model the 3D structure considering the −964G > A polymorphism (rs1632947) into HRE. [33,35]. The 5′TAAAAACAGGCAGTGC(G/A)TGAGCACTAGTGAGGG′3 (−980 to −948) sequence was used to construct a double-stranded type B (B-DNA) DNA, with the second complementary strand being constructed from the input sequence (named as −980r to −948r as a mention of reverse sequence). Both DNA molecules generated (one for the −964G allele and other for the −964A allele) are identical in terms of their structural conformation except for the nucleotide sequence differing at the −964 position. The B-DNA of DNA conformation was chosen for modeling of the HRE at the *HLA-G* promoter region because it is equivalent to the biologically predominant form of DNA in the body [41,42]. 

### 2.3. HIF1-HRE-964G/A Docking

Molecular docking is a method widely used in structural biology to explore the interaction of residues between two molecules. Once 3D structures of HIF1 and DNA (HRE964G/A) were obtained by molecular modeling, it was possible to proceed with protein-DNA docking using the HADDOCK v.2.4 server [43,44]. HIF1 has its DNA binding domain (DBD), bHLH, docked to its HRE in the promoter region of the *HLA-G* gene, emphasizing the existing polymorphism within the HRE. Thus, two protein-DNA complexes may be formed with the molecular docking technique using the same protocol, the HIF1-HRE-964G and HIF1-HRE-964A complex, which were named according to the allele present at the −964 polymorphic site within the HRE of the promoter region of the *HLA-G* gene.

In summary, HADDOCK is based on information from the interaction interface of the molecular complex predicted by experimental or computational approaches, which are introduced as ambiguous interaction restrictions (AIRs) to the direct docking. Such AIRs are defined through a list of residues that fall into two categories: active and passive [44], with a careful selection of which residues are active and which are passive, a step that is fundamental for the docking success.

Several DBDs have been studied in the literature by experimental methods, such as X-ray crystallography of protein-DNA complexes. This allows the identification of the DBD amino acids directly involved in the intermolecular bond with DNA bases or with sugar-phosphate backbone. HIF1 binds to HRE mainly through the N-terminal α-helices 1 present in its α (HIF1a) and β (ARNT) subunits. Important DNA binding residues within α-helices 1 for DNA interaction were introduced based on crystallographic structures belonging to the same bHLH-PAS family of HF1 transcription factors. These proteins are formed by two subunits that interact with each other, forming heterodimers that bind to the regulatory element present in several target genes, such as the HIF1 transcription factor [45]. The crystallographic structures of heterodimers that share the ARNT subunit have a globally similar architecture between them when linked to the regulatory element, as observed for the mouse crystals from the AHR-ARNT-DNA (PDB ID: 5V0L) [46], HIF1a/HIF2a-ARNT-DNA (PDB ID:4ZPR/4ZPK, respectively) [37], and NPAS3-ARNT-DNA (PDB ID: 5SY7) [47] complexes. These proteins present DNA-binding residues conserved among the bHLH family of transcription factors, such as S22, R23, A25, A26, R29, and R30 for the α subunit (HIF1a) and H94, E98, R101 and R102 for the β subunit (ARNT) (Supplementary material, Figure S4), which were selected as active residues for HIF1 (HIF1a-ARNT) to drive docking process of both molecular complexes, HIF1-HRE-964G and HIF1-HRE-964A. Active residues for DNA molecule were selected based on the consensus sequence of HRE 5’GCRTG’3 [48], in which the “R” position could be “G” (HIF1-HRE-964G complex) or “A” (HIF1-HRE-964A) allele. Passive residues were automatically selected by the server.

For the HIF1-HRE-964G complex, HADDOCK grouped 313 structures in 23 clusters, representing 78% of the 400 water-refined models that HADDOCK generated, whereas for the HIF1-HRE-964A complex, HADDOCK grouped 326 structures in 25 clusters, representing 81% of the 400 models refined in water. These clusters were classified according to their HADDOCK score (HS), with the more negative being better.

**Figure 2:**
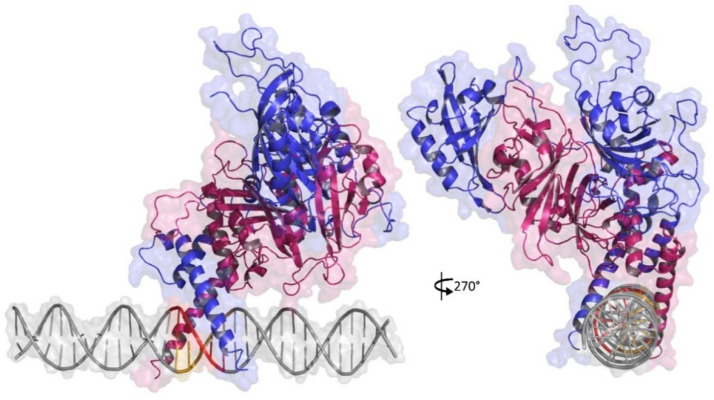
3D representation of similar docking pose obtained to HIF1-HRE molecular complexes generated by HADDOCK server. HRE in DNA double helix is highlight in red. Chain A is highlighted in reference to β subunit (ARNT) in blue and chain B is highlighted in reference to α subunit (HIF1a) in pink. Note: Author’s own image.

Top ranked poses which were clustered in the ten top clusters by HADDOCK server were analyzed for their structural similarity against a protein-HRE reference complex that belongs to the bHLH-PAS family of transcription factors: HIF2a-ARNT-HRE complex (PDB ID: 4ZPK). Their 3D structures were superimposed in PyMol v.2.4 to RMSD calculation (data not shown) to identify a pose with similar structure to the classic conformation of the bHLH-PAS family [45]. Most of the clustering poses presented HRE orientation at the HIF1 DBD different from expected; however, the second-best classified cluster for HIF1-HRE-964G (cluster 20) and the fourth-best classified cluster for HIF1-HRE-964A (cluster 2) presented the most similar poses to the reference complex. Poses of both clusters were similar with it one (Figure 2) and showed RMSD lower than the 2.0 Å tolerance level when aligned to the crystal, showing good structural similarity.

Physical-chemical characteristics from partner molecules define the affinity degree between them and are causally related to the intermolecular interactions in the complex. Then, energy functions derived from force fields and empirical terms are used to delineate correct and incorrect poses generated in the docking procedure by binding affinity estimation between molecules [49]. In turn, binding affinity between molecules is related to Gibbs free energy (ΔG). In analogy to any spontaneous process, the connection between two molecules only occurs when changes in the free Gibbs energy (ΔG) of the system is negative, indicating that the system has reached an equilibrium state with constant pressure and temperature [50]. Thus, the best poses generated in the docking process are those with negative intermolecular interaction energy values, as they indicate a probable biomolecular interaction [44,50]. HADDOCK orders the best poses based on their HADDOCK score (HS), which is a score function that consists of the linear combination of various energy terms. Then, a pose with the lowest HS is considered the most energetically favorable structure. The best-ranked poses for HS in clusters 20 and 2 were chosen as the final structure of the HIF1-HRE-964G and HIF1-HRE-964A complexes, respectively. Table 2 shows the values of the binding energy terms for the final structures of the HIF1-HRE-964G and HIF1-HRE-964A complexes generated by HADDOCK.

As illustrated in Table 2, the HS value for the HIF1-HRE complex presenting the −964G allele (HS: −163.53 kcal/Mol) was lower compared to the HIF1-HRE complex containing the −964A allele (HS: −147.90 kcal/Mol). This difference suggests that the HIF1-HRE-964G complex is more energetically favorable than the HIF1-HRE-964A complex. In addition, HIF1-HRE964G complex showed a higher value for the buried surface area in relation to the transcription factor bound to the HRE of the −964A allele, indicating a higher interaction interface between the partner molecules in the first complex. These results show that the presence of the A allele at the −964 polymorphic site in the HRE at *HLA-G* promoter region decreases the interaction between HIF1 and HRE.

In the last decades, the increased number of protein-DNA high-resolution structures deposited in the PDB along with the availability of advanced technologies to explore regulatory elements in DNA, such as ChIP-seq, have allowed a greater expansion of knowledge about the protein-DNA binding specificity [51,52]. Additionally, protein-DNA complex binding specificity can be understood by atomic interactions between amino acids and nucleotides including hydrogen bonds and van der Waals (VdW) contacts [53]. These interactions occur between protein side chains with sugar-phosphate backbone and with nitrogen bases in the major and minor grooves, in which the first interaction type is important for molecular complex stabilization and the second interaction type is responsible for specificity [54,55,56]. Since genetic variations at the regulatory elements may alter the specificity of protein-DNA complex binding, [57] the analysis of HIF1-HRE intermolecular binding pattern considering −964G > A SNP at the *HLA-G* promoter region is important to the understanding of the gene transcription regulation.

Intermolecular interaction analysis was performed using the DNAproDB server [58] and visualized in PyMol v.2.4. Both complexes are bound to their regulatory elements through hydrogen bonds and VdWs contacts formation between α-helix 1 residues of the HIF1 subunits with the nitrogen bases in the major DNA groove and with the sugar-phosphate skeleton. When HIF1 binds to HRE containing the alternative −964A allele at the −964G > A variation site, 11 hydrogen bonds and 87 VdW contacts stabilize the complex, while in the presence of the −964G allele, 16 hydrogen bridges and 81 VdW contacts were identified.

**Figure 3:**
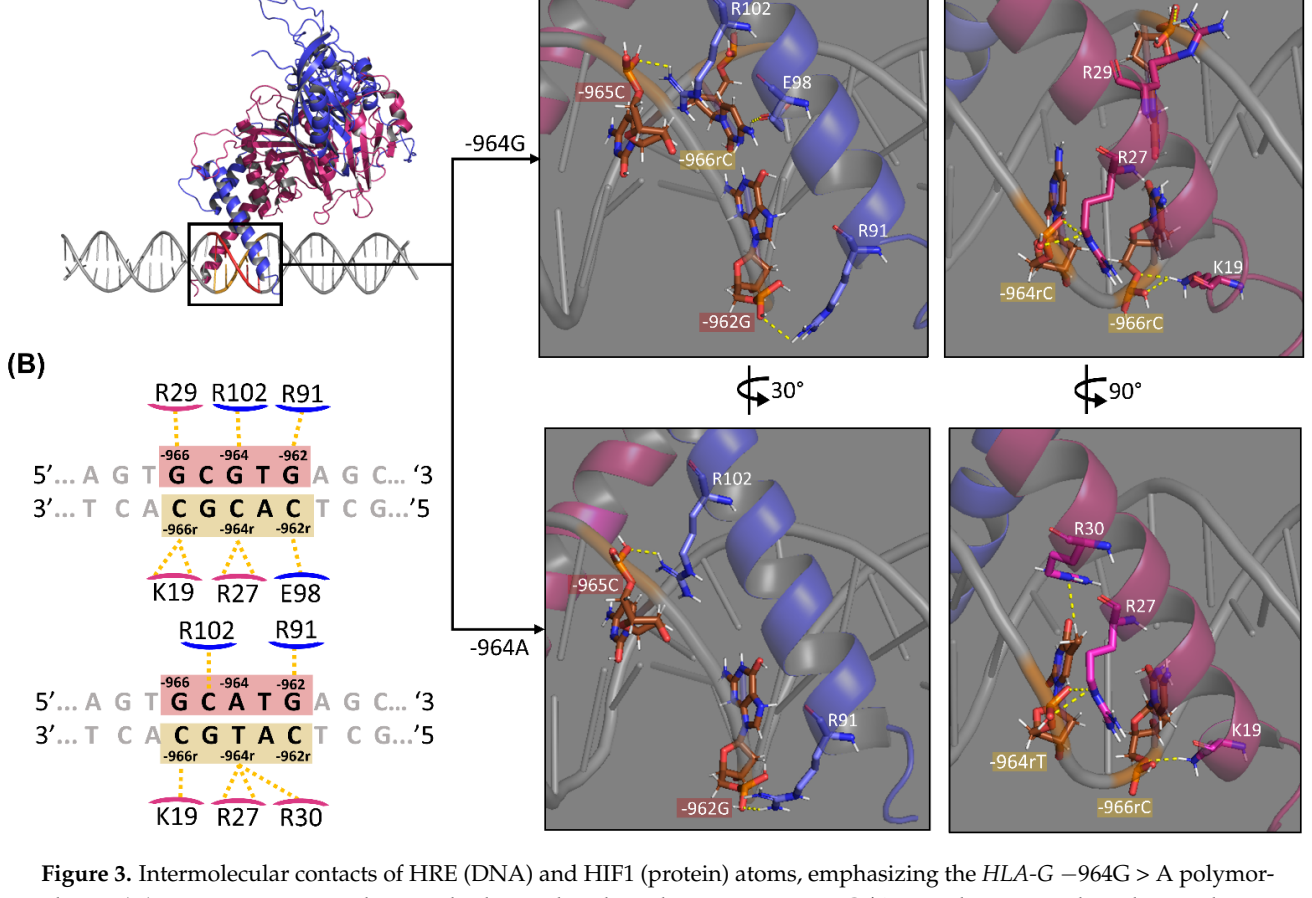
Intermolecular contacts of HRE (DNA) and HIF1 (protein) atoms, emphasizing the *HLA-G* −964G > A polymorphism. (**A**) 3D representation showing hydrogen bonds at the HIF1-HRE-964G/A complexes regarding the regulatory element. The central box is a 3D illustration of the HRE (highlight in red at the DNA structure, and its complementary sequence in sand) recognition by HIF1 bHLH domains and on the right are illustrated the details regarding hydrogen bonds formed between residue atoms of the ARNT (blue) and HIF1a (pink) with nucleotide atoms of the −964G/A alleles. Hydrogen bonds (distance between 2.5–3.2 Å) are indicated by yellow dotted lines. The red boxes indicate HRE nucleotides (5′-3′) and sand boxes indicate their complementary bases. Hydrogen atoms are colored in white, phosphate in orange, oxygen in red, and nitrogen in blue, while carbon is shown with different colors between partner molecules: DNA carbon in brown, HIF1a carbon in pink, and ARNT carbon in light blue. (**B**) 2D interaction diagram showing intermolecular contacts at the HIF1-HRE-964G/A complexes regarding the regulatory element. The red boxes indicate the *HLA-G* −964G/A alleles at the HRE, considering the base pair. Hydrogen bonds are indicated by yellow dotted lines. The red boxes indicate HRE nucleotides (5′-3′) and sand boxes indicate their complementary bases. Despite hydrogen bonds, VdW contacts at the HIF1-HRE complex also occur between residues-nucleotides (K128-966G, R102-964G, E98-963T, E98-962rC, S95-963T, H94-962G, and R91-962G in ARNT and R30-965C, R30-964rC, R30-963rA, and R23-965rG in HIF1a for the complex with the G allele, and R102-964A, R99-964A, E98-964A, H94-963T, and R91-963T in ARNT and R30-965C, R30-963rA, R23-965rG, and S22-966rC in HIF1a for the complex with the A allele, data not shown). Note: Author’s own image.

As expected, VdW contacts are more abundant in protein-DNA complexes than hydrogen bonds and stabilize the molecular complex as a whole, but hydrogen bonds are stronger non-covalent bonds [53,59,60]. The result analysis and discussion will be focused mainly on the amino acid-DNA contacts formed by the hydrogen bonds. In this context, eight hydrogen bonds are formed between HIF1 residues with HRE region in HIF1-HRE-964G, while six hydrogen bonds were observed in HIF1-HRE-964A, as shown in Figure 3.

Regarding the HIF1-HRE-964G complex, five residues are bound to HRE by sugar-phosphate skeleton atoms (R91 and R102 for ARNT and K19, R27, and R29 for HIF1a), while only the E98 residue binding directly to the bases. The K19 and R27 amino acids form two hydrogen bonds with −966rC and −964rC nucleotides, respectively. For the HIF1-HRE-964A complex, five bonds are formed between amino acid atom pairs and HRE-sugar-phosphate skeleton, which are shared between the two protein-DNA complexes, except for the hydrogen bond formed by the R30 of HIF1a with the nucleotide -964rT, which is observed only in the HIF1-HRE complex containing the −964A allele. Noteworthy, no HIF1 residue formed hydrogen bridges directly with the G/A variation site, only VdW forces act at the interaction of these bases (R102-964G for the HIF1-HRE-964G and R102-964A, R99-964A, and E98-964A for the HIF1-HRE-964A). In contrast, hydrogen bonds are formed with complementary bases of the −964G/A alleles on the reverse strand (3’-5’ direction) through the phosphate radical of the DNA chain (R27-964rT/C interaction in Figure 3), and this bond occurs independently from the −964G or A alleles.

The −964A allele altered HRE geometry in the complex HIF1-HRE-964A, resulting in interaction loss between partner molecules, such as the hydrogen bonds between K19-966rC and E98-962rC (Figure 3) observed to the complex with G allele. The number of hydrogen bonds formed between an amino acid and a nucleotide is directly proportional to the stability of the complex [60]; thus, the HIF1-HRE-964G complex is more stable than HIF1-HRE-964A. The E98 residue is highly conserved and shares a similar binding pattern among the bHLH-PAS family of transcription factors [37,46,47]. In addition, previous studies have shown that E98 is essential for the HRE interaction [61]. Thus, it is possible to infer that the −964A allele decreases HIF1-HRE binding specificity, as well as the stability of the molecular complex.

In addition to the residues shown in Figure 3, hydrogen bonds between amino acids and nucleotides neighboring HRE were identified interacting with nucleotide base atoms (H94-961rT, S22-968G, and S22-967T for the HIF1-HRE-964G complex and S22-967T and S22-968G for the HIF1-HRE-964A complex) and with sugar-phosphate backbone atoms (R101-961rT, K21-969A, and R29-967T for the HIF1-HRE-964G complex and K21-969A and R29-967T for the HIF1-HRE-964A complex). Overall, hydrogen bond analysis showed a decreased interaction of HIF1 with HRE in the presence of the *HLA-G* −964A allele when compared to the G allele, indicating that HIF1 binds in a more stable and specific manner with HRE in the presence of the −964G allele.

### 2.4. Protein-DNA Molecular Dynamic Simulation 

**Figure 4:**
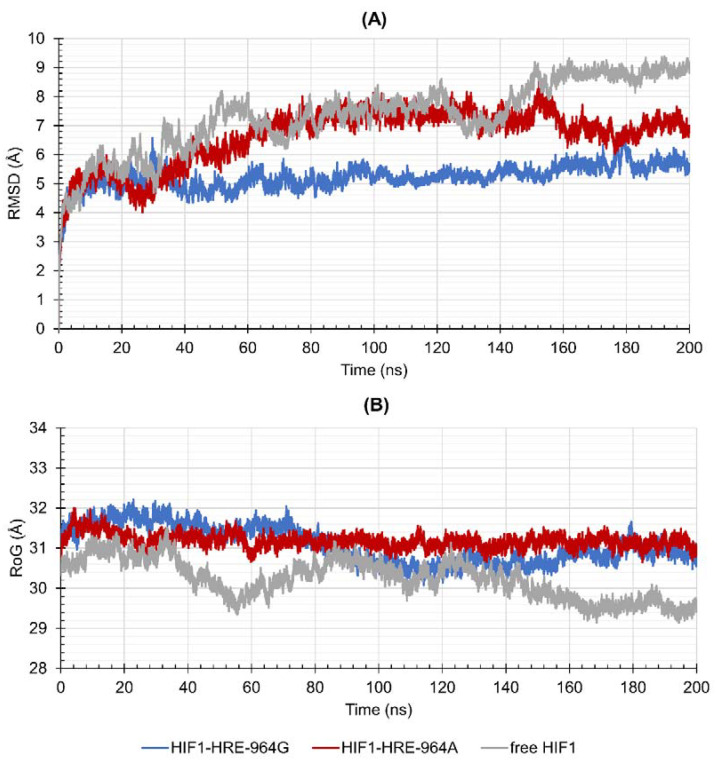
Values of (**A**) Root Mean Square Deviation (RMSD) and (**B**) Radii of Gyration (RoG) over 200 ns for the HIF1 transcription factor (HIF1a-ARNT) bound to the HRE according to the *HLA-G* −964G > A polymorphism in comparison with the free HIF1. Note: Author’s own image.

To verify the dynamic behavior of the protein-DNA complexes, we proceeded with the molecular dynamic simulation that evaluates complexes’ stability over time. Conformational variations of HIF1 complexed or not to HRE-964G/A were measured by Root Mean Square Deviation (RMSD), Radii of Gyration (RoG), and Root Mean Square Fluctuation (RMSF) during 200 ns of simulation. Figure 4 shows the results obtained for RMSD (A), and RoG (B) for backbone atoms (N, Cα and C) from HIF1 structures in relation to the initial structure of the transcription factor. According to the RMSD results (Figure 4A), unbound HIF1 tends to equilibrate close to 50 ns, remains without major movements for the next 100 ns (7.44 ± 0.46 Å), and remains equilibrated until the end of the trajectory (8.79 ± 0.22 Å). HIF1 bound to HRE with G allele reaches a level of equilibrium in the first 10 ns of simulation and remains without movements until the end of the trajectory (5.27 ± 0.35 Å), while in the presence of the −964A allele, HIF1 stabilizes at 60 ns (7.19 ± 0.35 Å) until the end of the 200 ns. RoG shows the compaction or expansion processes related to the polypeptide chain or DNA folding, indicating conformational change in the molecule, in which a higher degree of compactness means lower values of RoG. According to RoG, free HIF1 showed low structural compaction throughout its trajectory (29.15 ± 0.49 Å), acquiring a slightly folding shape at the end in relation to the initial structure. HIF1 bound to HRE showed low structural compaction throughout the trajectory, independently from the HRE allele, as observed in Figure 4B (RoG average of 31.08 ± 0.46 Å for HIF1-HRE-964G, and 31.18 ± 0.17 Å for HIF1-HRE-964A).

**Figure 5:**
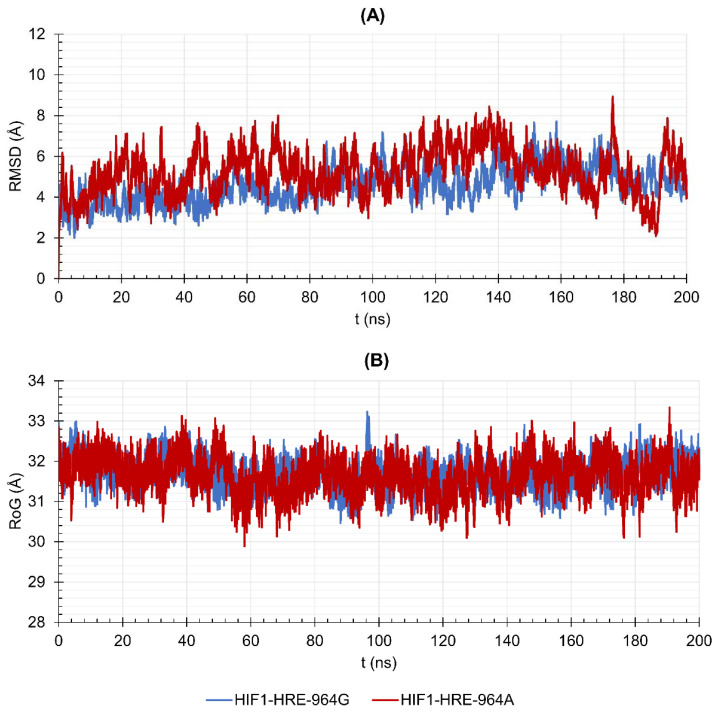
Values of (**A**) Root Mean Square Deviation (RMSD) and (**B**) Radii of Gyration (RoG) over 200 ns for the DNA double helix of HIF1-HRE complexes, according to the *HLA-G* −964G > A polymorphism. Note: Author’s own image.

HIF1 has higher structural stability when bound to the HRE containing the −964G allele when compared to the protein-DNA complex exhibiting the −964A allele. In addition, the HIF1-HRE with the A allele hits RMSD values like those of the free HIF1, reinforcing the hypothesis that HIF1 binding to HRE with the G allele is more stable. HIF1 folding did not differ between the two molecular complexes, which reached RoG values like that of the free transcription factor in the initial time; however, the free protein showed a slight compaction during the simulation, while the complexes remained unchanged, and accordingly, RoG results. RMSD and RoG for DNA were also analyzed (Figure 5A,B, respectively) showing a high flexibility for the two molecular complexes.

**Figure 6:**
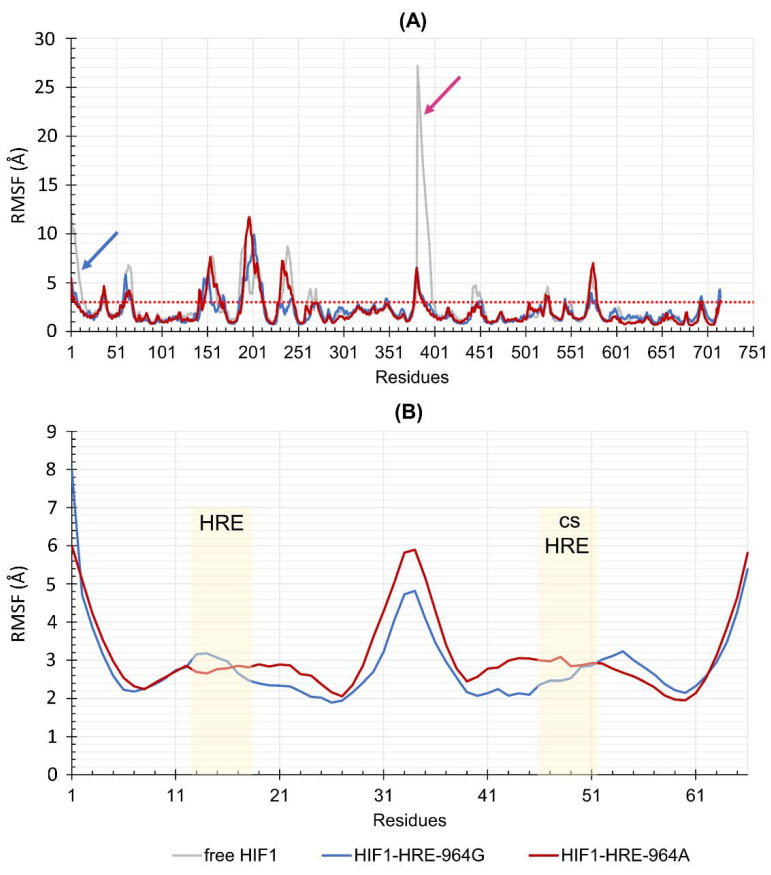
(**A**) RMSF values for the HIF1-HRE complexes according to the *HLA-G* −964G > A polymorphism. Residues 1 to 381 correspond to the ARNT subunit and residues 382 to 715 correspond to the HIF1a subunit. The dotted red line marks the amino acids with RMSF > 3.0 Å. The blue and pink arrows indicate the α-helices 1 of the ARNT (residues 1 to 18) and of the HIF1a (residues 382 to 400), respectively. (**B**) Comparisons of the DNA of the complex according to the *HLA-G* −964G > A polymorphism. Residues 15 to 19 correspond to the −966 to 962 hypoxia-responsive element (HRE) sequence and residues 48 to 52 correspond to the −966r to −962r complementary nucleotide sequence to the HRE. Both are highlighted in the beige boxes (the forward: HRE and the nucleotide sequence complementary to the HRE: csHRE). Each strand of DNA contains 33 bp. Note: Author’s own image.

RMSF describes residue position variation during the simulation, indicating the system flexibility. Figure 6A,B show the results of the RMSF for each HIF1 residue bound to HRE-964G/A in comparison to free HIF1 and to DNA chain nucleotides, respectively. HIF1 demonstrated a remarkably similar fluctuation pattern between the two protein-DNA complexes, differing only for some loop regions (regions above red line in Figure 6A), where the transcription factor linked to the HRE with the A allele presented higher fluctuation in these residues than the HRE with the G allele. The HRE binding to bHLH domain of the HIF1 has stabilized the α1 helix regions in the ARNT and HIF1α subunits (highlighted in the blue and pink, respectively, as shown in Figure 6) of the dimer for both HIF1-HRE-964G/A complexes, due to less fluctuation in these regions compared to the free protein. In fact, HIF1 must be complexed to its regulatory element at the promoter region of the gene to play its function; then, the absence of DNA anchorage to the HIF1 (free HIF1) results in high fluctuations at α-helix 1 of the DBD. In relation to the nucleic acid, fluctuations were observed mainly in the terminal nucleotides of the DNA double helix in both molecular complexes, while the protein binding site was considered stable (Figure 6B). The high fluctuation of the terminal nucleotides in both protein-DNA complexes justifies the high conformational flexibility, as observed for the RMSD, and RoG values for DNA.

**Figure 7:**
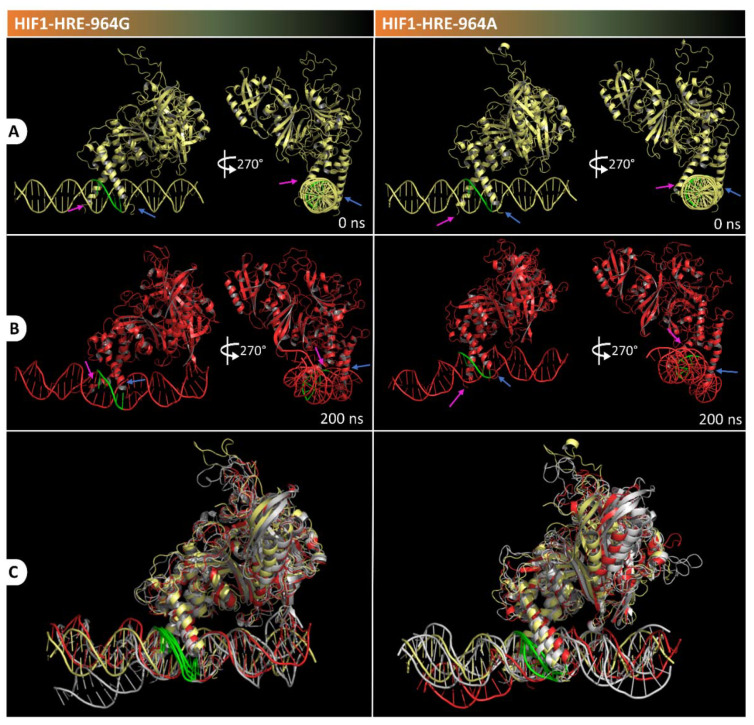
Visualization of trajectory snapshots at times 0 (**A**) and 200 ns (**B**) for the HIF1-HRE-964G and HIF1-HRE-964A complexes. In the Figure (**C**) is illustrated the structure superposition at specific times (0, 50, 150, and 200 ns) of the trajectory for the HIF1-HRE964G/A complexes, in which the initial structure is illustrated in yellow, the time final structure is illustrated in red, and other structures are illustrated in gray. The blue and pink arrows indicate the α-helix 1 region of the ARNT and HIF1a subunits, respectively. The HRE is highlighted in green in the DNA structure. Note: Author’s own image.

Visual inspection of the protein-DNA complexes was performed to visualize conformational changes during molecular dynamic simulation, as shown in Figure 7. Atomic coordinates from molecular complexes were extracted at specific times in the trajectory and were superimposed with initial structure (t = 0 ns) using the PyMol 2.4 software. Figure 6 shows snapshots captured at specific times in the trajectory for the HIF1-HRE-964G/A complexes. HIF1 remained bound to DNA in both complexes over the time and their secondary structures were preserved. HIF1 loop regions and the 5′-3′ terminal strand of the DNA double helix are regions with greater kinetics in the two molecular complexes analyzed, corroborating with the high RMSF values observed for such regions of the molecules.

Although the HIF1-HRE complex with the G allele presents more stability in comparison to the HIF1-HRE complex containing the A allele, RMSD and RoG values are still high. This may be due to the subunits of HIF1 (ARNT and HIF1α) that have intrinsically disordered regions (IDRs) located in the protein C-terminal regions and DBD N-terminal [62,63]. In this context, intrinsically disordered proteins (IDPs) and IDRs do not present a stable and unique conformation, which are able to adopt different conformations according to the molecular partner that interacts to play its biological function [64]. IDR properties are provided by their amino acid composition and can present different residue lengths accompanied by well-structured domains [65]. Thus, the acquisition of protein stability occurs when complexed to its molecular partner [66].

In vitro studies have shown that HIF1 bound to HRE located at the *HLA-G* promoter region (−966 HRE) does not induce gene transactivation and protein production under hypoxic conditions, but it acts in synergy with another HRE located at exon 2 (positions +281 sense and +291 antisense) [23]. The joint action of the two HREs positively regulates *HLA-G* gene transcription and protein production with the aid of cofactors, such as the CBP/p300 co-activator [21,67]. In turn, a DNA loop formation connects transcription factors and their target regulatory elements located away from the basal transcription complex, propitiating that the transcriptional process occurs independently from physical distance between HREs [68,69]. Thus, through the formation of a DNA loop, the HREs located at the promoter region and at exon 2 of the *HLA-G* gene become accessible to HIF1 binding in hypoxic conditions.

The absence of *HLA-G* exon 2 HRE and cofactors in our protein-DNA systems as well as the lack of the N-terminal transactivation domain (N-TAD) of the HIF1 [12,13], in which the cofactor CBP/p300 interacts with HIF1, may have influenced the higher values of RMSD and RoG, since these components support the transcription factor stability. On the other hand, HIF1-HRE complex containing the G allele was more stable than the other HIF1-HRE complex with the A allele according molecular dynamics results, corroborating previous experimental data, i.e., the HRE at the *HLA-G* promoter region alone is not able to induce the protein production in vitro; however, the G allele showed higher affinity for HIF1 than the A allele [23].

**Figure 8:**
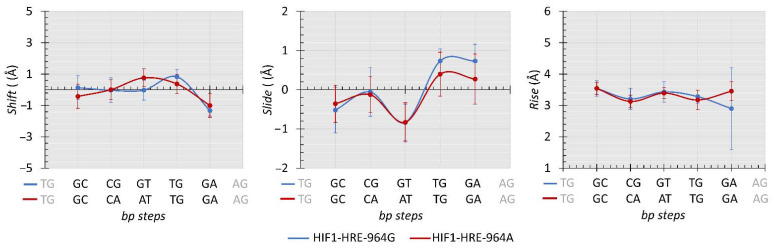
Average values (Å) comparisons regarding the shift, slide, and rise calculated for the HRE from HIF1-HRE-964G/A complexes trajectory atomic coordinates every 10 ns. Note: Author’s own image.

**Figure 9:**
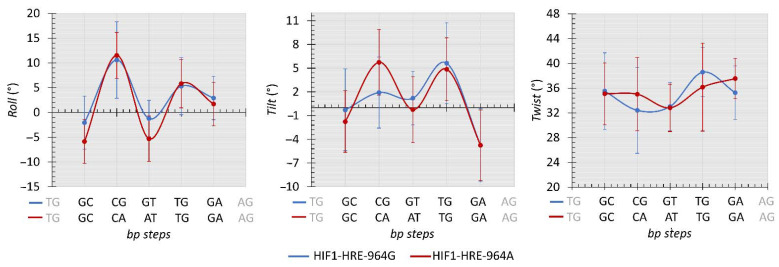
Average values (°) comparison of the roll, tilt, and twist calculated for the HRE from HIF1-HRE-964G/A complexes trajectory atomic coordinates every 10 ns. Note: Author’s own image.

Protein-DNA interaction induces changes in DNA double helix geometry due to DNA flexibility and the deformation caused by the protein in the nucleic acid structure is essential for molecular recognition, which is called indirect readout mechanism [70,71,72]. To analyze the differences in the conformational parameters of the 3D structure of the DNA double helix during the simulation of molecular dynamics of the HIF1-HRE complexes, inter base pair (bp) step parameters from protein-D trajectory atomic coordinates were calculated using Curves program [73]. The results are shown in Figure 8 and Figure 9 for the HRE in the analyzed molecular complexes.

Inter-bp step parameters are dependent of the DNA nucleotide sequence and are analyzed for each dinucleotide within the three chemical classes observed for the HRE: pyrimidine-purine (YR step), purine-purine (RR step), and purine- pyrimidine (RY step). These parameters comprise three translational distances (rise, shift, and slide) and three rotational angles (roll, twist, and tilt). Regarding the translational distances, molecular complexes showed remarkably similar values for most of the HRE dinucleotides in a manner independent from the −964G/A polymorphism (Figure 8).

In contrast, the presence of the polymorphism in the HRE caused slight deformations in the DNA double helix structure (Figure 9): (i) roll angle varied about 4 degrees in the first and third bp steps (RY and YR steps, respectively) of the HRE among the molecular complexes analyzed; (ii) tilt angle shifted 4 degrees in the second bp step, that is, in the dCG and dCA dinucleotides for the complexes containing the HRE with the G and A allele, respectively; (iii) twist angle showed a variation of approximately 3 degrees in the second and fourth bp step (TG dinucleotide, TGd): in relation to the second bp step, the HRE with the A allele has a higher value for the twist in relation to the complex with the HRE-964G, and then, in the fourth bp step, there is a conversion of values for the twist between these molecular complexes.

Generally, a structural parameter combination describes the deformations in the DNA double helix. For instance, dinucleotide curvature occurs preferably through the roll angle and may or may not be accompanied by translational movements on the slide [74,75,76]. Roll and slide show a negative correlation, where positive values for roll and negative for slide tend to cause a bending in the two stacked nucleotides towards the major groove, while negative values for roll and positive for slides cause a bend in the minor groove [76]. However, such a combination is not observed between roll and slide parameters for the HRE steps of our molecular complexes. In particular, patterns of side-by-side dinucleotides presenting positive and negative roll angles result in the production of a linear conformation of the double helix [77], inferring that HIF1 when bound to the HRE does not cause a curvature in the double helix, as shown in Figure 9 for most HRE steps.

Roll and twist rotational angles present higher flexibility and contribute to the curvature magnitude of the nucleic acid then tilt angle [72,75]. Among the three chemical classes observed for the HRE, YR steps (second and fourth bp-step in Figure 9) presented higher values for the roll and twist compared to the other bp steps, corroborating previous studies [77,78], except for the CGd of the regulatory element containing the G allele for the twist. In fact, the 5 ‘and 3’ ends of the HRE with the −964A allele are more flexible than the middle of the regulatory sequence, whereas for the HRE with the −964G allele, higher flexibility is observed for the 3 ‘end given by the fourth step in relation to the twist angle. This difference in the complex flexibility for the YR steps referring to the twist angle between analyzed complexes may be due to the presence of the −964G/A *HLA-G* SNP into the HRE, since the twist angle is more strongly affected by neighboring bases than the other conformational parameters [78]. The HIF1-HRE interaction may present a different indirect read out mechanism, irrespective of the *HLA-G* −964G > A polymorphism.

The tilt rotational angle does not substantially contribute to the DNA curvature and deformations in the double helix resulting from this angle are more energetically costly than other inter bp parameters [75,79]. Positive tilt values cause an opening of base pairs stacked on the strand, which high values cause steric clashes between these base pairs resulting in a penalty for the structure energy terms [72,80,81]. Thus, deformations caused by tilt angle in the bp steps generate local conformations that may affect DNA interaction with biological molecules, such as transcription factors. Again, YR steps are more flexible for tilt compared to the other HRE steps, as shown in Figure 9; however, the second CAd step of the HRE with the A allele showed higher values compared to the CGd of the HRE-964G. The presence of the A allele at the HRE generates a DNA double helix conformation that is less energetically favorable. To supply this energy penalty caused by the tilt angle, an increase in the rise parameter is required for the bp-steps [72]; however, no difference is observed for the rise in the CAd step of the HIF1-HRE-964A complex and the energy cost is not fixed. Furthermore, the tilt DNA double helix deformation in the HRE-964A may have influenced its interaction with HIF1, decreasing the stability of this molecular complex, as previously mentioned regarding the results of the HIF1-HRE-964A molecular dynamic simulations.

In conclusion, HIF1 interacts in a more stable and favorable manner with the HRE containing the *HLA-G* −964G allele when compared to the HRE containing the −964A allele. A limitation to understand the role of HIF1 on the transcription regulation of the *HLA-G* gene using computational molecular modeling techniques is the presence of other HRE motifs (exon 2 HRE), besides the one at the promoter region [23], demanding further analyses. Notwithstanding, the identification of the differential behavior of *HLA-G* −964G/A alleles at the atomistic level may explain the differential production of the immune checkpoint HLA-G molecule under hypoxic conditions. The understanding of the *HLA-G* gene regulation under hypoxic conditions may be particularly useful in conditions, in which the expression of the molecule may be advantageous for example pregnancy and transplantation, as opposed to conditions associated with harmful effects of the HLA-G expression, such as in cancer disorders.

## 3. Materials and Methods

### 3.1. HIF1 Dimer 3D Structure Modeling

For HIF1 quaternary 3D structure modeling, the crystal of mouse HIF2 dimer (PDB ID: 4ZPK with3.6 Å of resolution; chain A represents β subunit and chain B represents α subunit of HIF2 complex) was retrieved from Protein Data Bank (PDB) (https://www.rcsb.org/, accessed on 20 January 2020) and it was chosen as template to perform homology modeling. Missing regions in the crystal were filled by PDB Reader tool on the CHARMM-GUI server [82]. Primary structures of the HIF1 subunits, HIF1a (α) and ARNT (β), were retrieved from UniProt in fasta format, with UniProt IDs Q16665 for human HIF1a residues 15-349 and P27540 for human ARNT residues 87–470. These primary sequences were aligned against crystal chains according to their correspondents α and β subunits, using the Clustal X software [83], and then, the HIF1 dimer was modeled and refined using MODELLER v.9.24 [38,40]. The resulted models were evaluated for their template structural similarity by root mean square deviation (RMSD) of Cα atoms using align command with five cycles of outlier rejection (cycles = 5) of the PyMol v.2.24 software, and after, the model with the lowest RMDS value was evaluated according to the protein stereochemical and physical-chemical properties, using the following Model Quality Assessment Programs (MQAPs): PROCHECK [84,85], Verify 3D [86], ERRAT [87], and Qualitative Model Energy Analysis (QMEAN) [7].

### 3.2. DNA Modeling

We generated 3D structural models of DNA from the HRE sequence 5′GCGTG′3 present in the *HLA-G* promoter region at positions −966 and −962 (hg19_Chr6 position reference: 29794642-29794674), containing the G or A alleles of the −964G>A variation site (rs1632947) [34], along with 14 flanking nucleotides (5′TAAAAACAGGCAGTGC(G/A)TGAGCACTAGTGAGGG′3, referring to the −980 to −948 nucleotides), using the x3DNA v.2.4 software (LU, OLSON, 2008).

### 3.3. HIF1-HRE-964G/A Molecular Docking

The High Ambiguity Driven DOCKing HADDOCK webserver v.2.4 was used to drive protein-DNA dockings [43,88]. HADDOCK uses biochemical and biophysical interaction data of partner molecules, which are introduced as ambiguous interaction restrictions to guide the docking process by active and passive residue selection [44]. HIF1 was directly docked onto HRE consensus sequence containing the −964G or −964A allele. Active residues selected to HIF1 (HIF1a-ARNT) were S22, R23, A26, A27, R29, and R30 for the α subunit (HIF1a) and H94, E98, R101, and R102 for the β subunit (ARNT), which were conserved among the members of the bHLH-PAS protein family [37,46,47], and to DNA, −966G, −965C, −964G/A, −963T, and −962G, which correspond to the HRE sequence. Passive residues were automatically selected around active residues by the server. 

Input parameters for the HADDOCK server remained in the standard mode, with the exception of the following parameters: (i) the dielectric constant (epsilon), which was set to 78 for it0 and it1 due to the high DNA charge; (ii) the initial temperature for the third cooling step of the temperature-accelerated dynamic (TAD) with the fully flexible interface set to 300, (iii) the factor for the time interval in the TAD set to 4, (iv) the number of heating steps for the 300 K phase set to 750, and (v) the sampling parameters were changed to 10,000/400/400 for rigid body (it0), simulated annealing (it1), and water refinement (W) stages, respectively [89,90]. In each docking stage, poses were classified according HADDOCK score (HS) [44]. Protein-DNA interactions were analyzed with the aid of the DNAproDB server [58].

### 3.4. Molecular Dynamic Simulations

Molecular dynamic simulations (MD) were performed to the free HIF1 and protein-DNA complexes using the GROMACS v.2019 package. The simulations for the free protein were performed using the all-atom AMBER-99SB force field [91]; for protein-DNA complexes, the same force field was used with the parameters referring to the DNA modified according to the AMBER-Parmbsc1 [92]. Both systems were built in a cubic box solvated with SPC/E water with a minimum of 14 Å of distance between the edge and any atom of the protein or molecular complexes. General charge of the systems was neutralized with the addition of Cl^−^ and K^+^ ions to reach the total concentration of KCl to 150 mM. An energy minimization for 0.1 nanoseconds (ns) was carried out to eliminate bad contacts between the atoms, followed by an equilibration step with number of particles, volume, and temperature constants (NVT) and another with number of particles, pressure, and temperature constant (NPT). MD simulations were carried out at constant pressure and temperature (1 atm and 300 K) per 200 ns [93]. Deviations in the structural analyses, such as RMSD, root mean square fluctuations (RMSF), and radii of gyration (RoG), were done from MD trajectories. 

Conformational parameter differences of the DNA double helix structure during the molecular dynamic simulation of the HIF1-HRE-964G/A were assessed with Curves software [73]. All the helical parameters, such as the translational distances (rise, shift, slide), and the rotational angles (roll, tilt, twist) of the DNA were calculated from protein-DNA trajectory atomic coordinates every 10 ns, starting at time t = 0 ns (21 structures in total). The results are presented as mean and standard deviation.

### 3.5. 3D Structure Visual Analysis

The 3D structures of free protein as well protein-DNA complexes and their intermolecular interactions were visualized using PyMol Molecular Graphics System v.2.4 [94].

## Figures and Tables

**Table 1:** Quality assessment of the HIF1 model compared to template.

MQAPs	4ZPK	HIF1
PROCHECK–Ramachandran Plot (1)		
Most favorable regions	83.10	87.20
Additional allowed regions	15.70	11.10
Generously allowed regions	0.80	0.90
Disallowed regions	0.40	0.80
ERRAT ARNT-HIF1/2a (2)	66.67–58.43	76.16–70.86
Verify-3D (3)	41.77	68.87
QMEAN (4)	−2.44	−2.77

Reference values: (1) Best: >90% in most favored regions; (2) Good high resolution structures generally produce values >95% (<2.5 Å), and for lower resolutions (2.5 to 3.0 Å), the average overall quality factor is around 91%; (3) Good: ≥80%, regular: <80%, worse: <65%; (4) z-score values close to 0 indicate that the model has quality compatible with high-resolution structures with similar size, and values lower than −4.0 indicate that the model has low quality. MQAP: Model Quality Assessment Program; 4ZPK: HIF2 crystal structure; HIF1: HIF1 refined homology model; Total residues number: ZPK: 610 aa; HIF1: 715 amino acids.

**Table 2:** Binding energy terms values comparison between the HIF1-HRE-964G and HIF1-HRE-964A complexes generated by HADDOCK.

Terms	HIF1-HRE-964G	HIF1-HRE-964A
HS (a.u.)	−163.53 (−152.70 ± 6.60)	−147.90 (−140.60 ± 4.60)
Evw (kcal/mol)	−84.98 (−87.70 ± 3.90	−84.37 (−81.00 ± 3.90)
Eelec (kcal/mol)	−536.02 (−478.20 ± 38.90)	−435.78 (−425.80 ± 23.10)
Eair (kcal/mol)	3.68 (12.10 ± 13.00)	4.70 (7.80 ± 3.00)
Edesol(kcal/mol)	28.29 (29.40 ± 2.40)	23.15 (24.80 ± 1.50)
BSA (Å)	2195.65 (2241.2 ± 122.40)	2050.90 (2065.30 ± 86.90)

HS: HADDOCK score; EVdW: van der Waals (VdW) energy; Eelec: Eletrostatic energy; Edesol: desolvatation energy; Eair: restrains violation energy; BSA: Buried Surface Area; a.u.: arbitrary units; statistics were calculated from the four best poses HS ranked for cluster 20 to the HIF1-HRE-964G, and for cluster 2 to the HIF1-HRE-964A.

## Data Availability

Not applicable.

## Supplementary Materials

The following are available online at https://www.mdpi.com/article/10.3390/ijms222313046/s1.
