# Supplementary material for: Structural Characterization of the Interaction of Hypoxia Inducible Factor-1 with Its Hypoxia Responsive Element at the −964G > A Variation Site of the HLA-G Promoter Region
Source: Int J Mol Sci. 2021 Dec 2;22(23):13046. doi: 10.3390/ijms222313046 (PMC8657931; doi:10.3390/ijms222313046)
Supplement: Supplementary file 1 [file ijms-22-13046-s001.zip › ijms-1429313-supplementary.pdf]

International Journal of
Molecular Sciences

MDPI

Supplementary materials

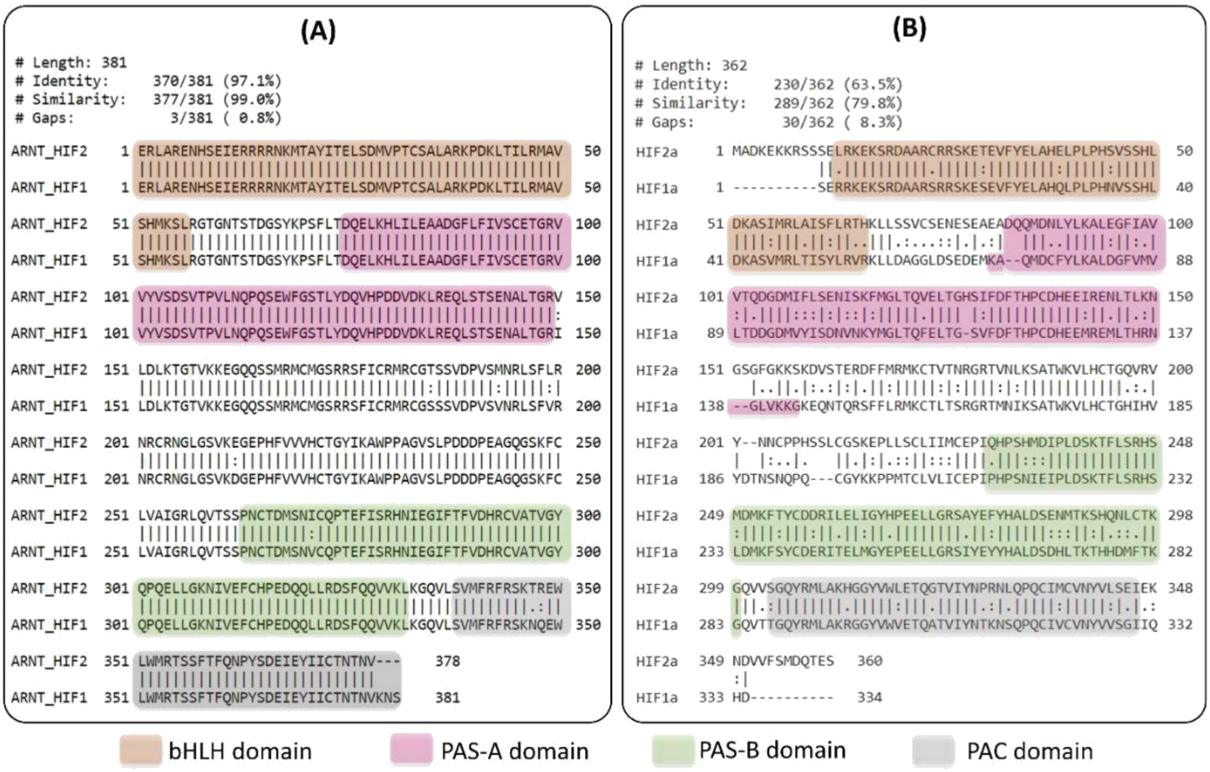

Figure S1. Global alignment between the primary sequences of (**A**) β (ARNT) and (**B**) α (HIF1/2a) subunits of HIF1 (human) and HIF2 (mouse) transcription factors performed using the EMBOSS Needle software. bHLH: basic-Helix-Loop-Helix; PAS-A/B: Per-ARNT-Sim A/B; PAC: Per-ARNT-Sim associated C-terminal).

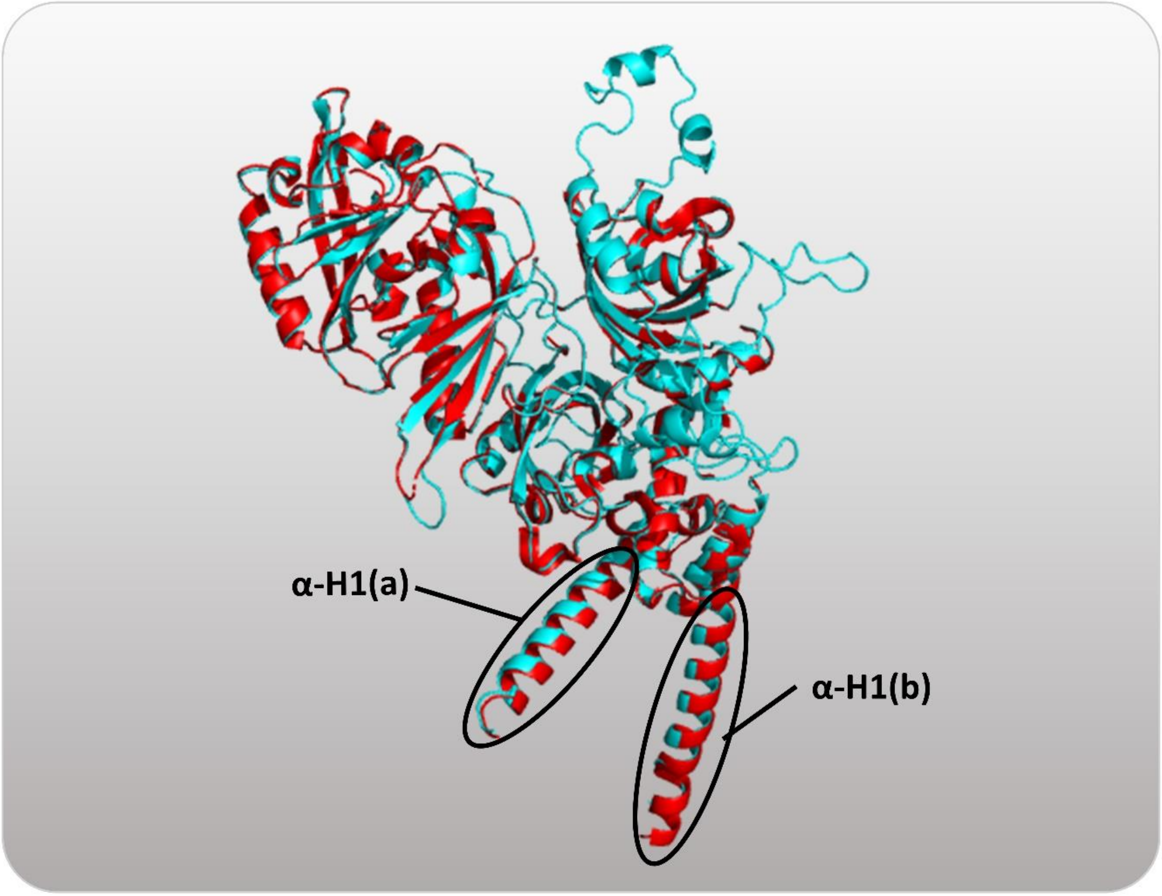

Figure S2. 3D structure alignment between homology generated HIF1 model 8 and HIF2 crystal (PDB ID: 4ZPK). The structure in red corresponds to the crystal (template) and in aqua-green corresponds to model 8 with a RMSD value of 0.396Å. α-H1 (**a**) highlight region refers to the α1-helix of the HIF1a/HIF2a, and α-H1 (**b**) refers to α1-Helix of the ARNT. Alignment was performed using align command (cycle = 5) of the PyMol v.2.4 software.

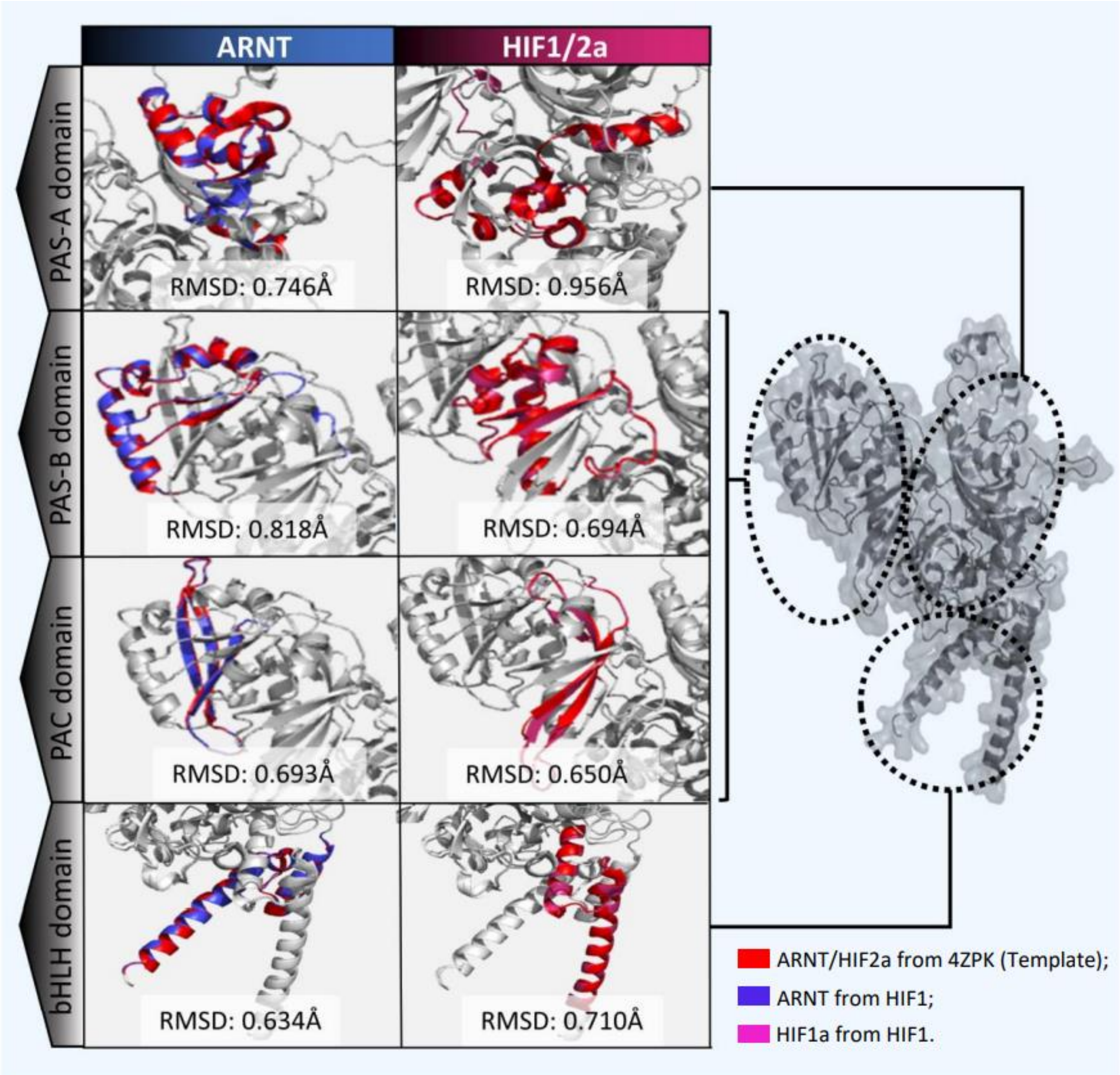

Figure S3. Protein domains alignment between HIF1 model 8 and 4ZPK crystal (template). On the left, protein motifs were aligned against each other in the model 8 and the 4ZPK crystal using PyMol align command without outlier rejection (cycles = 0) to the α-subunits (HIF1/2a) and β-subunits (ARNT). On the right, protein 3D structure representation with motifs regions highlighted with a dotted circle. bHLH: basic-Helix-Loop-Helix; PAS-A/B: Per-ARNT-Sim; PAC: Per-ARNT-Sim associated C-terminal.

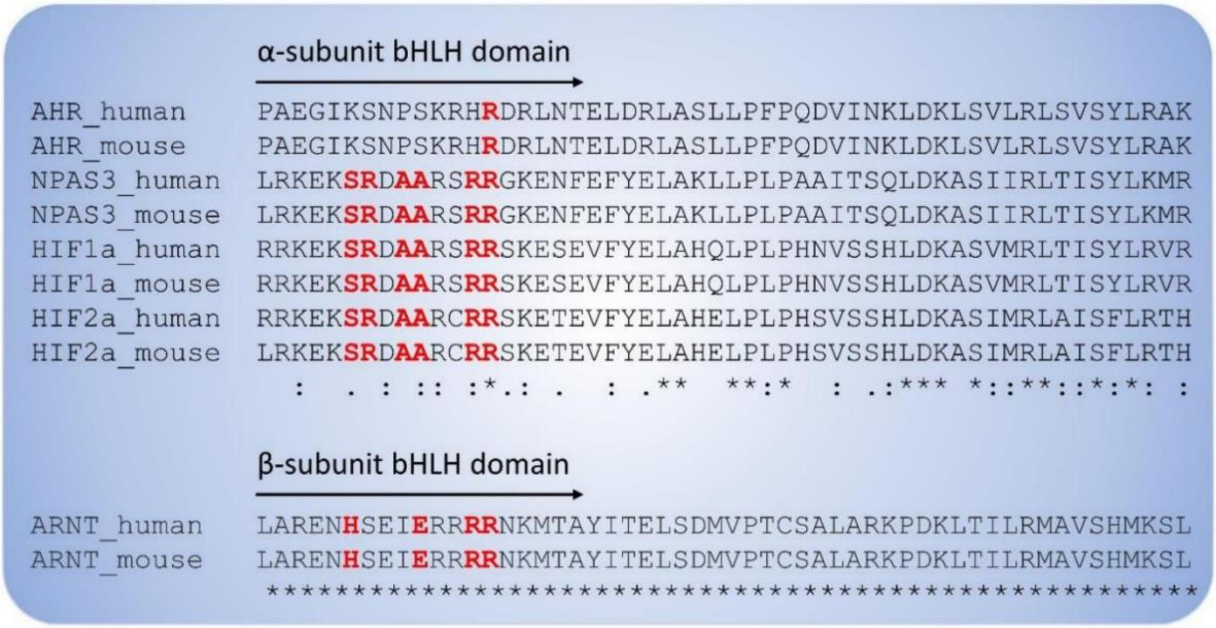

Figure S4. Comparison of the bHLH DNA-binding domain among ARNT-binding proteins from mouse and human. Multiple alignment between α-subunits that interact with the ARNT (β-subunit) to form DNA-binding heterodimers performed by Clustal Omega v.1.2 server. bHLH sequences were retrieved from UniProt database-https://uniport.org (UniProt IDs: P35869 to AHR, Q8IXF0 to NPAS3, Q99814 to HIF2a and Q16665 to HIF1a of human α-subunits; P30561 to AHR, Q9QZQ0 to NPAS3, P97481 to HIF2a and Q61221 to HIF1a of mouse α-subunits; P27540 to human ARNT and P53762 to mouse ARNT). In the Figure, DNA-binding residues highlighted in red in the α and β subunits. bHLH: basic-Helix-Loop-Helix; AHR: Aryl hydrocarbon receptor; NPAS3: neuronal PAS domain proteins 3; HIF1/2a: Hypoxia inducible factor 1/2a; ARNT: Aryl hydrocarbon receptor nuclear translocator.
